# Gene Expression Profile of Peripheral Blood Lymphocytes from Renal Cell Carcinoma Patients Treated with IL-2, Interferon-α and Dendritic Cell Vaccine

**DOI:** 10.1371/journal.pone.0050221

**Published:** 2012-12-03

**Authors:** Benita Wolf, Adrian Schwarzer, Anik L. Côté, Thomas H. Hampton, Thomas Schwaab, Eduardo Huarte, Craig R. Tomlinson, Jiang Gui, Jan L. Fisher, Camilo E. Fadul, Joshua W. Hamilton, Marc S. Ernstoff

**Affiliations:** 1 Medical Oncology Immunotherapy Group, Section of Hematology/Oncology, Dartmouth Hitchcock Medical Center, Lebanon, New Hampshire, United States of America; 2 Department of Gastroenterology, Hepatology and Infectious Diseases at University Hospital of Tübingen, Germany; 3 Hannover Biomedical Research School, Experimental Paediactric Haematology and Oncology, Hannover, Germany; 4 Department of Urology, Roswell Park Cancer Institute, Buffalo, New York, United States of America; 5 Department. of Medicine, Immunotherapy Program, Norris Cotton Cancer Center, Dartmouth Medical School, Lebanon, New Hampshire, United States of America; 6 Department of Pharmacology/Toxicology, Dartmouth Medical School, Hanover, New Hampshire, United States of America; 7 Section of Biostatistics and Epidemiology, Department of Family and Community Medicine, Dartmouth Medical School, Lebanon, New Hampshire, United States of America; 8 Veterinary Molecular Biology, Montana State University, Bozeman, Montana, United States of America; 9 Marine Biological Laboratory at Woods Hole, Woods Hole, Massachussetts, United States of America; UCLA, United States of America

## Abstract

Lymphocytes are a key component of the immune system and their differentiation and function are directly influenced by cancer. We examined peripheral blood lymphocyte (PBL) gene expression as a biomarker of illness and treatment effect using the Affymetrix Human Gene ST1 platform in patients with metastatic renal cell carcinoma (mRCC) who received combined treatment with IL-2, interferon-?-2a and dendritic cell vaccine. We examined gene expression, cytokine levels in patient serum and lymphocyte subsets as determined by flow cytometry (FCM). Pre-treatment PBLs from patients with mRCC exhibit a gene expression profile and serum cytokine profile consistent with inflammation and proliferation not found in healthy donors (HD). PBL gene expression from patients with mRCC showed increased mRNA of genes involved with T-cell and T_REG_-cell activation pathways, which was also reflected in lymphocyte subset distribution. Overall, PBL gene expression post-treatment (POST) was not significantly different than pre-treatment (PRE). Nevertheless, treatment related changes in gene expression (post-treatment minus pre-treatment) revealed an increased expression of T-cell and B-cell receptor signaling pathways in responding (R) patients compared to non-responding (NR) patients. In addition, we observed down-regulation of T_REG_-cell pathways post-treatment in R vs. NR patients. While exploratory in nature, this study supports the hypothesis that enhanced inflammatory cytotoxic pathways coupled with blunting of the regulatory pathways is necessary for effective anti-cancer activity associated with immune therapy. This type of analysis can potentially identify additional immune therapeutic targets in patients with mRCC.

## Introduction

Renal cell carcinoma (RCC) accounts for approximately 5% of all malignancies. [1], [2] Currently available treatment options for patients with metastatic disease (mRCC) rarely result in a cure. Although IL-2 based immunotherapy carries significant acute toxicity, it is the only treatment to date, that results in durable complete remissions in about 5% of patients. [3]–[5] Due to the lack in understanding of both the complex interaction between the tumor and the host immune system, and the host of factors that lead to response to immune therapy, it cannot be as yet predicted who will benefit from IL-2. While initial studies suggested that tumors expressing high levels of carbonic anhydrase IX (CAIX), a hypoxic inducible factor, respond better to IL-2 therapy, a prospective trial failed to confirm this. [6], [7].

Gene expression profiling, an important tool to study complex biological processes, can map involved genes to known pathways and thus is useful for hypothesis generation. [8], [9] In this study we used microarray technology to highlight differences in gene expression of the peripheral blood lymphocyte population (PBL) in mRCC patients compared to healthy controls. We further evaluated PBL gene expression patterns for pre and post-therapy from patients treated with a combined immunotherapy regimen which resulted in a 50% clinical response rate. Since the PBL includes most of the cellular subsets thought to participate in the immune surveillance of cancer (T cells, B cells, and NK cells), this analysis can provide a comprehensive snapshot of immune status using a simple tool and potentially complement measurements made on tumor infiltrating lymphocytes. [8], [10]–[12].

## Materials and Methods

### Treatment of Patients and Isolation of PBLs and Serum

Protocol D0238 (Phase II Clinical Trial with IL-2, IRN-α2a and autologous dendritic cell (DC) tumor vaccination) and Leukapheresis Protocol D9726 were approved by the Dartmouth College’s Committee for the Protection of Human Subjects (CPHS).

As previously described, 18 informed and consented patients (mean age 61 years, 5 females, 13 males) with advanced mRCC were treated within a phase II clinical trial with IL-2, IFN-α2a and autologous dendritic cell (DC) tumor vaccination. [13] All procedures are conducted according to the principles expressed in the Declaration of Helsinki. Briefly, peripheral blood monocytes were cultured *ex vivo* into mature autologous tumor lysate loaded DCs and frozen for future vaccines. Treatment consisted of 5 cycles of one intranodal vaccination of DCs, 5-days of continuous intravenous infusion of IL-2 (18MiU/m^2^) and 3 subcutaneous injections of IFN-α2a (6MiU) every other day. PBLs for microarray analysis and flow cytometry, and serum samples for cytokine analysis were obtained pre-treatment and post-treatment (3 weeks after the 2^nd^ of 5 vaccination cycles and 2 weeks after completion of the 2nd cycle of IL-2 and IFN-α2a therapy). PBLs were isolated from pheresis product by elutriation fractionation. Since age is known to affect the status of the immune system, we identified 9 older healthy donors who signed our CPHS approved consent and underwent leukapheresis. [14], [15].

### PBL mRNA Extraction and Gene Microarrays

Total RNA was isolated from PBLs using RNAeasy kit (Quiagen, Valencia, CA) according to the manufacturer’s instruction. Hybridizations were performed according to Affymetrix guidelines (Affymetrix, Santa Clara, CA) at the Dartmouth College Microarray Shared Resource. Biotin-labeled cDNA was generated from 5.5 µg of total RNA and hybridized to the GeneChip® Human Gene 1.0 ST Array. The stained array was scanned at 532 nm using an Affymetrix GeneChip Scanner 3000.

Microarray analysis and description was carried out according to Minimum Information About a Microarray Experiment (MIAME) guidelines. The dataset has been deposited in NCBI’s Gene Expression Omnibus (http://www.ncbi.nlm.nih.gov/geo/query/acc.cgi?acc=GSE34465) and is accessible through GEO Series accession number GSE34465.

### Flow Cytometry of Lymphocyte Subpopulations

Lymphocyte subpopulations were characterized by standard five-color flow cytometry and analyzed with FlowJo software. [16] Intracellular staining for IL-4 (R&D) and IFNγ (BD Pharmingen) was done following cell fixation and permeabilization and intra-nuclear staining for FoxP3 (BioLegend FoxP3 kit) was done as previously described. [13].

### Luminex Assay for 27 Serum Cytokines

Luminex® fluorescent bead technology was used to measure serum levels for 27 cytokines [IL-1β, IL-2, IL-4, IL-5, IL-6, IL-7, IL-8, IL-9, IL-10, IL-12 (p70), IL-13, IL-15, IL-17, basic fibroblast growth factor, Eotaxin, granulocyte colony-stimulating factor, granulocyte macrophage colony-stimulating factor, IFN-γ, IP-10, MCP-1 (MCAF), MIP-1α, MIP-1 β, platelet-derived growth factor (PDGF), RANTES, tumor necrosis factor-α, vascular endothelial growth factor]. Serum of 7 patients PRE (3 R, 4 NR) and 8 patients POST (4 R, 4 NR), as well as 5 healthy controls were analyzed according to manufacturer’s protocol. [13] Due to limitations in patients material serum could not be analyzed for all patients.

### Response Criteria

To determine how gene expression, lymphocyte subsets and serum cytokine levels related to clinical outcome, we categorized patients according to clinical response using RECIST 1.0 criteria. [17] Overall objective clinical response rate for the total of 18 patients treated was 50% with three complete responses. The 17 pre-treatment patients included in our analysis were 9 R and 8 NR. Post-treatment, 13 patients (8 R, 5 NR) could be included in our microarray analysis, due to limited availability of PBLs for this time point.

### Microarray Data Analysis & Statistics

Chip-quality was controlled with Affymetrix® expression console™. As the computational basis, open source R software package (http://www.r-project.org) and tools from Bioconductor (www.bioconductor.org) were used. Probe level data of raw Affymetrix cell intensity (CEL) files were normalized and summarized using robust multichip average (RMA). When discussing gene expression, we refer to summarized probe set expression values. Our analysis follows practices suggested by The MicroArray Quality Control (MAQC) project augmented with a novel exploratory use of k-means analysis in the context of pathway analysis. [18]–[20] The MAQC recommends selection of genes of interest based on a fold change cutoff combined with a non-stringent p-value. We identified genes as differentially expressed if they achieved: A) a fold difference between conditions of 1.4 or greater and B) a t-test significance <0.05 assuming unequal variance. For comparison of serum cytokine levels as well as surface marker expression, Student t-Test or Whitney rank sum test was used to test for differences between groups. P<0.05 was considered significant and values were expressed as mean ± StD.

### Biological Interpretation

#### Pathway analysis

Canonical pathways were generated through the use of Ingenuity Pathway Analysis (IPA, Ingenuity Systems®, http://www.ingenuity.com) and Kyoto Encyclopedia of genes and genome (KEGG) database. IPA provides computational algorithms to identify and generate significant biological networks and pathways that are particularly enriched with identified genes of interest. The significance of the association between the data set and the canonical pathway is measured by: 1) a ratio of the number of molecules from the data set that map to the pathway divided by the total number of molecules that map to the canonical pathway. 2) Fisher’s exact test for p-value calculation, determining the probability that the association between the genes in the dataset and the canonical pathway is explained by chance alone. We also performed independent Gene set enrichment analysis (GSEA) for up-regulated and down-regulated gene lists to test for enrichment of involved pathways. [21] KEGG and IPA derive their data from published work. Since there is a recognized lack of consistency between these two databases, [22] we focused on pathways that are enriched in both platforms or in one platform as well as in GSEA.

### Clustering Analysis

Nonhierarchical unsupervised cluster analysis: K-means analysis was used to identify candidate paths as follows: we identified 190 KEGG paths with at least 5 genes that mapped to our microarray probes. We used k-means as implemented in R to identify two distinct sample clusters based on the gene expression values in each pathway. Finally, we then used p values from Fisher’s exact test to assess the strength of the association between k-means clusters.

In addition, we used Manhattan distance to perform hierarchical unsupervised clustering. The degree of similarity using the Manhattan distance tends to become larger for pairs of vector data that are less similar, and outlying data are slightly emphasized. [23].

### Confirmation of Selected Genes with Quantitative Real Time PCR

After reverse transcription of 1 ug total RNA using Quiagen reverse transcription kit (Quiagen, Valencia, CA), quantitative real time PCR was performed using the iCycler iQ Real-Time detection System (Bio-Rad Laboratories, http://www.bio-rad.com) and specific primer assays from Quiagen (Quiagen, Valencia, CA, Table S1). GAPDH was used as house-keeping gene. PCR analysis confirmed the gene chip expression data (Table S2).

### Analysis of Gene Expression Data, PBL Subsets and Serum Cytokine Levels

Analysis of gene expression (GE), serum cytokine levels (SCL) and lymphocyte subsets (LS) was performed for disease effects by comparing results for HD vs. mRCC patients PRE (GE: n = 9 vs. 17, SCL: n = 5 vs. 7, LS: n = 9 vs. 17). For analysis of treatment effects mRCC patients PRE vs. mRCC patients POST results were compared (GE: n = 17 vs. 13, SCL: n = 7 vs. 8, LS: n = 17 vs. 17). We also examined differentially expressed genes for R vs. NR PRE and POST as well as R PRE vs. R POST and NR PRE vs. NR POST (R PRE: n = GE:9, SCL:3, LS:8, R POST: n = GE:8, SCL:4, LS:8, NR PRE: n = GE:8, SCL:4, LS: 9, NR POST: n = GE:5, SCL:4, LS:9).

## Results

### Difference in Gene Expression, Serum Cytokine Level and Lymphocyte Subsets of mRCC Patients vs. Healthy Controls

**Figure 1:**
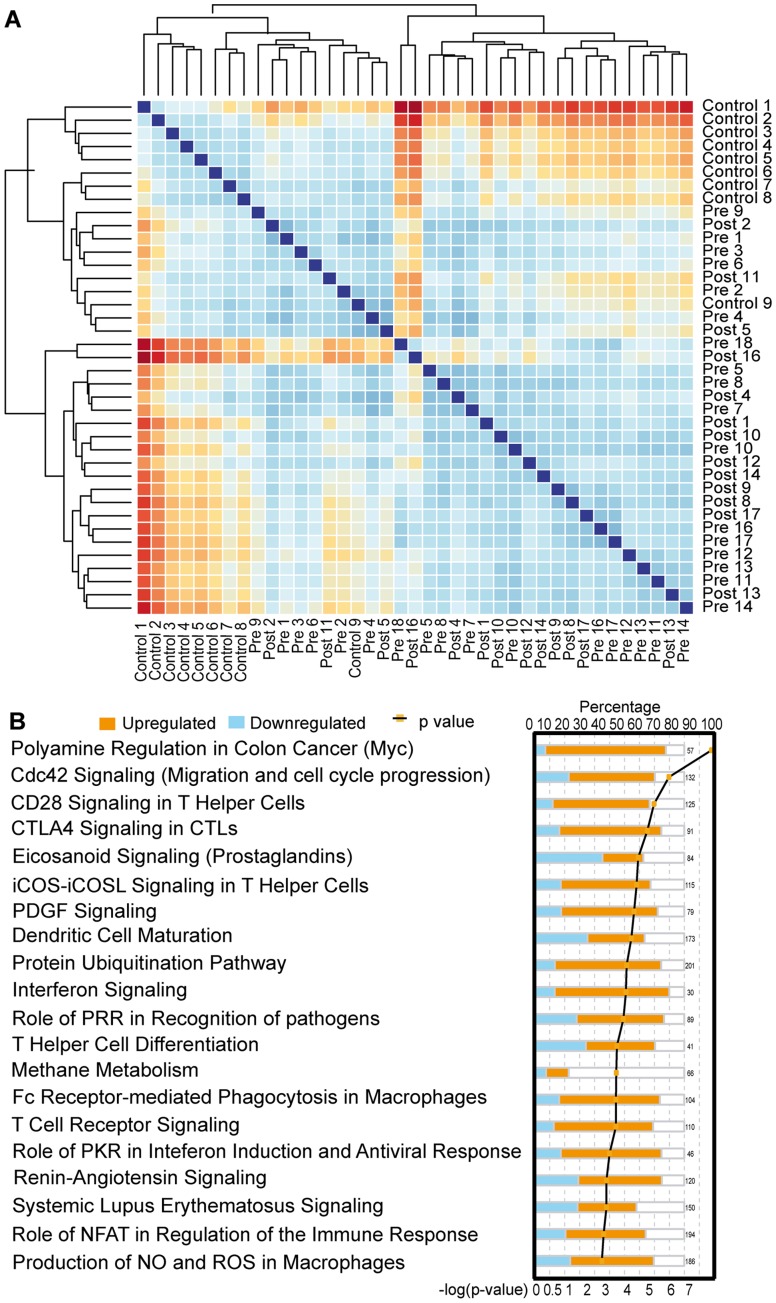
Comparison of gene expression of PBLs of mRCC patients and healthy controls following normalization of microarray expression data. ** A:**Microarray Data: The gene-expression profiles of PBLs from all patients (pre- and post-treatment) and from healthy donors (control) were compared by unsupervised hierarchical clustering using Manhattan distance after mean centering for definition of hierarchy. Shown is a correlative or adjacency matrix. A gene expression profile for each chip is calculated and compared with those of all other chips. The chips with similar gene expression profile cluster together. Red denotes a large distance between gene expression of arrays; dark blue means no distance (diagonal line). The largest distance can be detected for the comparison Control1 versus mRCC patient 16 Post. **B:** Ingenuity Pathway Analysis (IPA): A list of highly altered pathways after comparing gene expression between healthy controls and patients pre-treatment. Percentage of genes (of all known involved genes) altered in our dataset is shown on the top axis. Orange bars indicate up-regulation of genes and blue bars indicate down-regulation of genes. The bottom axis shows the log p-value that indicates how significantly the identified genes are associated to the pathway. IPA identified pathways reflecting an overall activation of the immune system as well as an alteration of T- and B-cell signaling pathways.

The gene expression profiles of PBLs from patients PRE and POST and from healthy donors (control) were all compared by unsupervised hierarchical clustering using Manhattan distance after mean centering for definition of hierarchy. This cluster analysis revealed that patients with mRCC, both PRE and POST, have a gene expression profile signature that is clearly distinct from HD (Figure 1A). To identify key biological pathways altered in mRCC patients, we used IPA. This platform identified pathways reflecting an overall activation of the immune system (Figure 1B). The most enriched pathway in PBLs was the polyamine regulation in colon cancer pathway (p = 2×10^−7^), which revealed the transcription factor *Myc* as over-expressed in lymphocytes from mRCC patients suggesting an overall increase in gene transcription, as *Myc* may regulate up to 15% of all genes. [24].

**Figure 2:**
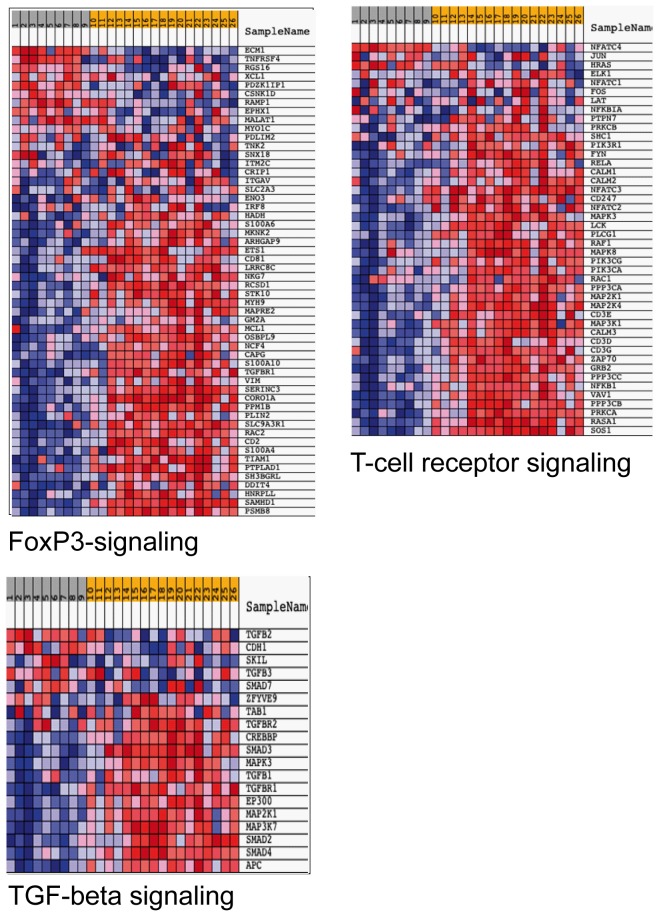
Comparison of gene expression of PBLs of mRCC patients and healthy controls following normalization of microarray expression data. Gene Set Enrichment Analysis (GSEA) was performed for 17 mRCC patients PRE (yellow background, #10–26) vs. 9 healthy controls (grey background, #1–9). GSEA uses a Kolmogorov Smirnov statistic to explore unique gene signature of small groups of genes within a data set. Shown are representative pathways that were up-regulated in the mRCC samples (p<0.05).

Additional identified key pathways are involved in regulating effector T cell activation. Supervised hierarchical clustering for T-cell and B-cell receptor associated genes for all arrays revealed a clear clustering of patients from healthy controls (Figure S1). On closer inspection, the pathways that are up-regulated in association with mRCC reflect a gene expression profile of enhanced transcriptional activity and overall activation of innate and adaptive immune pathways. The overall up-regulation of genes in the cytotoxic T lymphocyte-associated antigen 4 (CTLA4) signaling pathway (p = 5×10^−5^ Figure 1B) suggests active inhibitory pathways in CD4 effector T cells or a potential T_REG_ cell involvement in mRCC patients. Among the top 5 most significantly affected canonical pathways, we noted an increased expression of genes associated with the cdc42 signaling pathway but an overall decrease in genes associated with the eicosanoid signaling pathway (Figure 1B) for mRCC PBLs. In addition, GSEA showed activation of FoxP3 targets, as well as activation of T-cell receptor signaling and TGFß-signaling for patients when compared to HD (Figure 2).

A pattern of immune system activation in mRCC patients is further supported by results from our serum-cytokine analysis of 27 immune relevant cytokines and chemokines. In serum from healthy donors (n = 5), only 4 cytokines had detectable levels in every healthy control (IL-6, IFN-γ, PDGF and RANTES). 15 cytokines had higher levels in patients PRE serum compared to serum from healthy controls (IL-1ra, IL-4, IL-6, IL-7, IL-8, IL-12, IL-13, G-CSF, IFN-g, IP-10, MIP-1a, MIP-1b, PDGF, RANTES, VEGF). 10 of these contrasts reached statistical significance (Table 1). Up-regulation of PDGF-signaling was further supported by the IPA results (Figure 1B). The significantly up-regulated genes are important in T-cell, as well as B-cell development, activation and proliferation (IL-1ra, IL-4, IL-6, IL-7 and RANTES), angiogenesis (PDGF, VEGF), inflammation (MIP-1b, VEGF) or are stimulated by IFN gamma (IP-10). Th1 as well as Th2 specific cytokines were up-regulated, reflecting a state of immune activation.

**Table 1:** Serum cytokine (27 plex) assay results.

		Healthy control n = 5	Patients pre treatment n = 7	
	Limit of detection [pg/mL]	Mean [pg/mL] ± SD	Mean [pg/mL] ± SD	t-test p value
IL-1ra	1.4	13.2±18.1 (in 2/5)	129.9±38.5	0.111
IL-4	0.5	<0.5	2.1±0.7	0.004*
IL-6	1.1	3.6±0.5	13.1±9.4	0.072
IL-7	0.5	3.6±3.9 (in2/5)	10.3±2.7	0.009*
IL-8	0.5	3.4 (in 1/5)	7.7±5.8	0.166
IL-12	0.5	<0.5	2.6±1.9	0.326
IL-13	2.1	<2.1	9.3±6.6	0.296
G-CSF	1.1	4.4±6.1 (in 2/5)	17.1±7.2	0.017*
IFN-γ	1.0	7.0±3.1	16.6±4.0	0.025*
IP-10	6.5	8.8 (in 1/5)	106.9±49.9	0.002*
MIP-1a	2.4	<2.4	3.4±1.4	0.027*
MIP-1b	1.1	6.0 (in 1/5)	83.4±24.6	<0.001*
PDGF bb	1.0	1100.8±764.3	6205.6±2242.2	<0.001*
RANTES	1.2	2624.8±772.9	5594.1±908.3	<0.001*
VEGF	0.5	7.8±7.6 (in 3/5)	123.4±95.9	0.010*

* p<0.05.

Ten cytokines showed significant up-regulation in patients vs. healthy controls; 5 additional cytokines showed a clear increase in mRCC patients, but due to low sample size and high standard deviations these contrasts did not reach significance. IL-1b, IL-2, IL-5, IL-9, IL-10, IL-15 and IL-17 had detectable values in very few of the 16 tested samples and were thus excluded from the analysis and table.

Lymphocyte subset analysis by flow cytometry showed that mRCC patients had an up-regulation of CD4^+^ T-cells (47.6±15.6% HD vs. 61.7±12.7% mRCC, p<0.05) and a simultaneous down-regulation of cytotoxic CD8^+^ T-cells (48.3±14.7% HD vs. 35.8±13.0% mRCC, p<0.05). Furthermore regulatory T(T_REG_) cells, defined as CD25^+^FoxP3^+^ T cells (CD3+CD4+) were significantly up-regulated in mRCC patients (1.3±0.5% vs.2.9±1.0%, p<0.001). [13].

The properties of circulating T regulatory cells of the same patients and healthy volunteers were evaluated separately combining phenotype examination, DNA methylation analysis and global transcriptome analysis. [25] Data from all three types of analysis, microarray analysis, serum cytokine levels and cell subsets based on FCM show patterns which are indicative of immune activation including more inhibition and suppression of the immune system in mRCC patients compared to healthy donors.

### Treatment-related Effects on Gene Expression

To explore treatment-related effects on gene expression in mRCC patients, in addition to unsupervised hierarchical clustering (Figure 1A), a supervised cluster analysis was performed that revealed broad similarities in the gene expression profiles of patients’ pre and post-treatment PBLs (Figure S2). We could not discern a specific set of genes or key pathways that could discriminate a treatment effect.

### Gene Expression Profiles and Clinical Outcome

We investigated the data set for a gene expression profile that could predict response to immune therapy by comparing the gene expression of responders and non-responders pre-treatment PBL. Supervised analysis of patient PRE data revealed broad similarities in both groups of patients, as responders and non-responders clustered together (Figure S3A). Furthermore, there were no clear differences in the gene expression profile of patients POST PBL that could be correlated with clinical outcome (Figure S3B).

**Figure 3:**
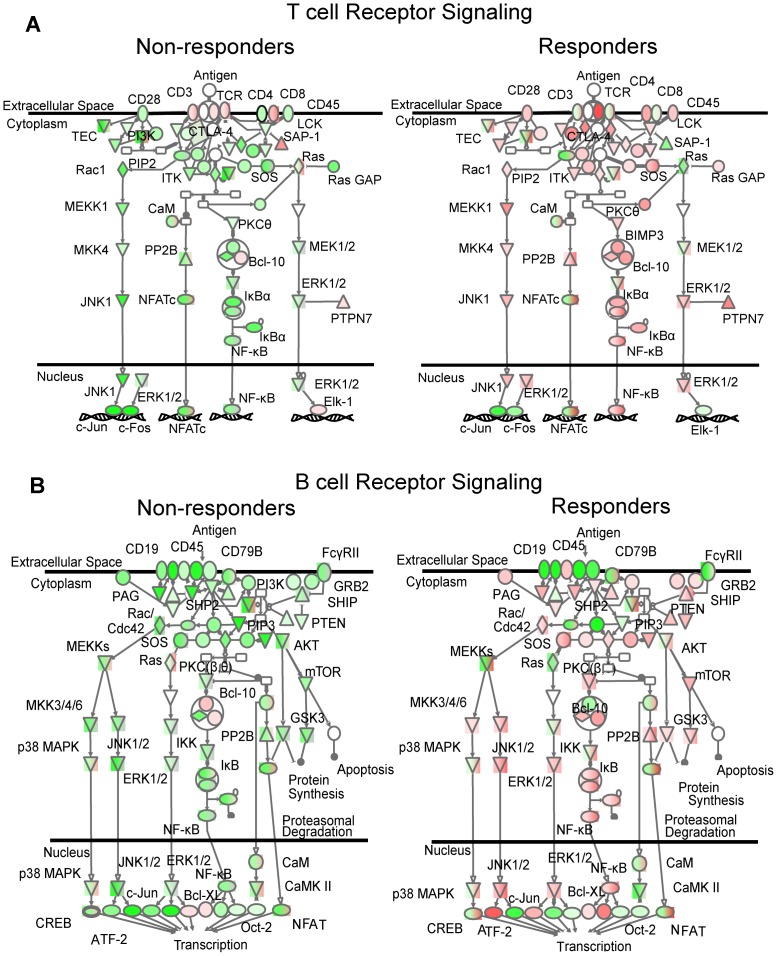
Separate comparison of gene expression post-treatment versus gene expression pre-treatment for responding and non-responding patients. Ingenuity pathway analysis suggests differences in regulation of T-cell (**Figure 3A**) and B-cell (**Figure 3B**) receptor signaling. Most of the molecules in the B-cell as well as in the T-cell receptor pathway are down-regulated for non-responding patients upon immunotherapy (POST <PRE), whereas gene expression of the same molecules is up-regulated for responding patients (POST >PRE). Red: up-regulation, Green: down-regulation.

To interrogate the data further we analyzed lists of differentially expressed genes between R POST vs. R PRE (n = 8 vs. 9) as well as NR POST vs. NR PRE (n = 5 vs. 8) with IPA. This analysis revealed that T-cell (Figure 3A) and B-cell (Figure 3B) receptor pathways were differentially regulated in responders and non-responders upon treatment. Responding patients, as a group, exhibit up-regulation of genes enriched for these pathways (Figure 3 A and B, right) while non-responding patients seemed to down-regulate those genes (Figure 3 A and B, left). This is again complemented by flow cytometry results, where the subset of TH2 T cells (IL4^+^ % of CD4^+^) significantly increases with treatment (PRE mRCC: 4.2±3.0% vs. POST mRCC: 8.9±7.1%, p<0.05) and this effect is due to the subset of responding patients (PRE R: 4.8±3.7% vs. POST R: 12.0±8.1%, p<0.05) not the non-responding patients (PRE NR: 3.6±2.2% vs. POST NR: 4.8±1.5%, ns).

**Figure 4:**
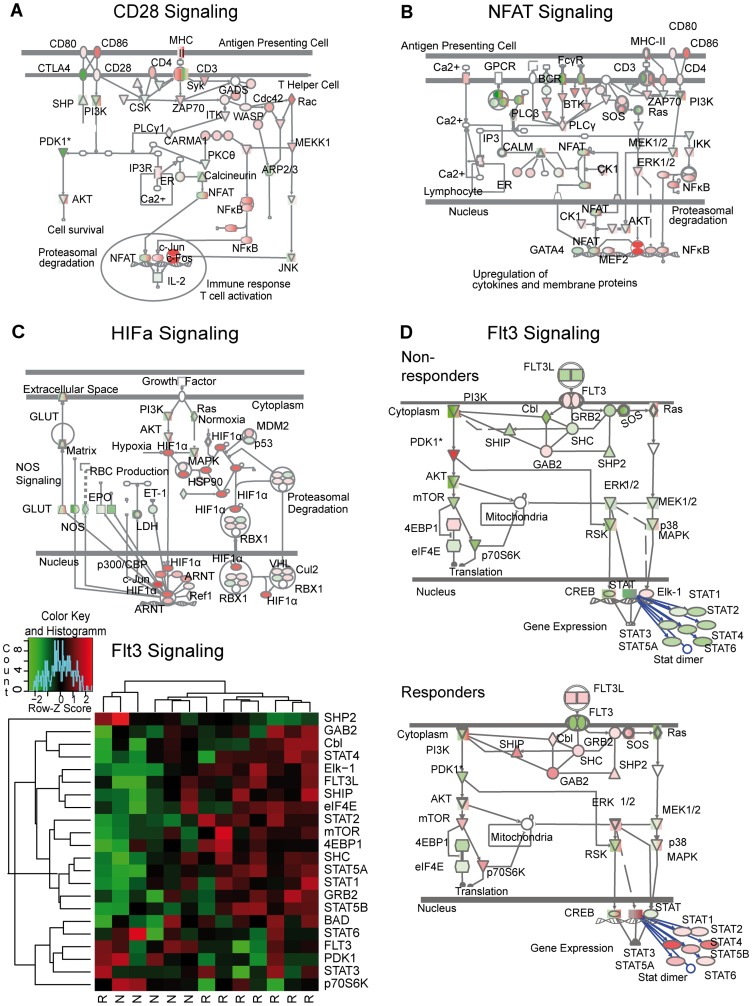
Comparison of treatment related changes in gene expression for responding versus non-responding patients (POST minus PRE). Immune therapy leads to activation of stimulatory pathways in responding subjects. Examples of activated pathways are shown. The CD28 co-stimulatory, nuclear factor of activated T-cell (NFAT) and HIFα pathways were relatively up-regulated (Figure 4A–4C) for responding subjects. Another key pathway (IPA) differentially regulated in R vs. NR PBLs was the Flt3 signaling pathway (fms-like tyrosine kinase receptor-3; CD135) in which genes were overall down-regulated in non-responding patients but up-regulated in responding patients (Figure 4D). To determine whether the genes picked by ingenuity software are able to discriminate between responding and non-responding patients, we performed supervised (selected genes) clustering analysis of genes important for Flt3 signaling. This clearly grouped responders and non-responders in 2 distinct groups (Figure 4D), strengthening the IPA-result. Supervised clusters of pathways in 4A–4C led to similar results (data not shown).

Furthermore, genes associated with the CD28 and NFAT signaling pathways were differentially expressed in responding and non-responding subjects in POST vs. PRE comparison. The CD28 co-stimulatory, nuclear factor of activated T cell (NFAT) and HIFα pathways were relatively up-regulated in responding subjects upon immunotherapy (Figure 4A–4C). Another key pathway (IPA) differentially regulated in R vs. NR PBL was the Flt3 signaling pathway (fms-like tyrosine kinase receptor-3) where genes were overall down-regulated in non-responding patients but up-regulated in responding patients (Figure 4D) as a result of treatment. Unsupervised clustering of genes from this pathway grouped POST responders and POST non-responders in 2 distinct groups (Figure 4D, bottom, left), suggesting that stimulating this pathway may be required to achieve a good clinical response.

Our analysis of possible response related genes also revealed that our immunotherapy regimen altered the CTLA-4 status of responding patients. Whereas patients PRE compared to healthy controls showed an activation of CTLA-4 signaling, this activation appeared to be reversed in responding patients following treatment. CTLA-4 pathway related genes in responders POST PBL are down-regulated compared to POST PBL of non-responding patients (Figure S4).

Subset analysis by FCM and cytokine multiplex results also revealed differences between responding and non-responding patient samples. The proportion of T_REG_ cells in the PBL, a key inhibitory cell subset, increases as a result of treatment for the NR patients compared to the R patients. (R PRE: 1.5±1.0% vs. R POST: 2.7±1.0%, NR PRE: 2.3±0.8% vs. NR POST: 7.0±2.8%). [13] Cytokine multiplex results showed an overall treatment related increase in serum levels of anti-angiogenic factor IP-10 (PRE 126±71 pg/ml vs. POST 521±244 pg/ml, p = 0.002, (Figure S5). Responding patients had higher levels of IP-10 in PRE serum than non-responding patients (R 139±41 pg/ml vs. NR 82±44 pg/ml, p = 0.08). Baseline IP-10 levels have been shown to correlate with clinical outcome in renal cell carcinoma and hepatocellular carcinoma. [16], [26] IP10 was the only serum cytokine level measured which differed based on treatment or response.

## Discussion

Global immune dysfunction develops in many patients with advanced tumor burden and alterations of the immune response may compromise immunotherapeutic approaches. [27]–[30] To investigate this further, we interrogated the gene expression profile of immune cells in the peripheral blood compartment. In this study, to corroborate microarray data, we measured the mRNA of selected genes independently by quantitative real-time PCR. We also quantified serum cytokine levels by enzyme-linked immunosorbent assay (ELISA) based multiplex analysis and determined the proportions of key lymphocyte cell subsets by flow cytometry analysis.

Our data show that the gene expression profile of PBLs in patients with mRCC compared to healthy volunteers reflects an inflammatory process with opposing signals: an increased expression of genes associated with T-cell activating pathways such as T- and B-cell receptor signaling, as well as heightened expression of genes involved in regulatory pathways such as CTLA4. This is reflected in altered lymphocyte subsets in mRCC patients as well as in increased serum cytokine levels. The overall gene expression of patients post-treatment vs. patients pre-treatment did not reveal relevant changes. This may reflect that the effects of mRCC on immune status include pro-inflammatory signals as well as signals that can inhibit anti-tumor responses. Our findings did not reveal any clear predictive value in the gene expression profile of patient pre-treatment PBL that could be correlated with outcome. However, analysis of POST minus PRE changes in gene expression in responders and non-responders demonstrated differential regulation of regulatory and inhibitory pathways in these two cohorts of patients and is supportive of the importance of therapeutic targeting of such pathways. These findings underscore the potential utility of gene expression signatures associated with disease-specific immune pathways to identify immune therapeutic targets in patients with cancer.

Twine et al. have reported disease-associated differences in the gene expression profile of peripheral blood mononuclear cells (PBMCs) from RCC patients compared to PBMCs of healthy volunteers. [9] Similar to our observations they report an induction of inflammatory-related genes. Correlation of fold changes of the 132 m-RCC related genes identified in the Twine dataset to fold changes for our data set revealed no similarities (Figure S6), although clustering analysis applying the Twine gene set, clearly discriminated our patients and healthy controls. The differences in gene expression may be related to the source of cells used for the two studies, PBMCs in the Twine study as compared to PBLs used in our study, and may also relate to the pool of healthy donors used for comparison in each study. [31] A meta-analysis of 47 studies emphasizes the global predicting role of systemic inflammatory response for relapse free survival in RCC. [32].

In our study, further pathway analyses with Ingenuity revealed an up-regulation of genes belonging to B-cell receptor and T-cell receptor signaling pathways in mRCC patients compared to healthy donors. The detected changes of genes in these pathways are supported by the documented importance of T-cell function, cell number, and specific T-cell functional pathways in the mRCC literature. [27], [33], [34].

While we observed increased levels of cytokines in the serum of patients with mRCC vs. healthy donors, [13] we were not able to demonstrate a corresponding increased expression in peripheral blood lymphocyte cytokine genes (data not shown). This suggests that the cytokine production may originate from another source (e.g. tumor infiltrating lymphocytes, RCC, endothelial cells, macrophages) or that the clearance from circulation of these cytokines is in some way diminished in patients with mRCC. The increase of cytokine levels could as well be due to an increase release of cytokines from internal compartments. [35].

We found treatment related up-regulation of 15 cytokines/chemokines including interferon IFN-γ inducible protein 10 (IP-10), Interferon gamma (IFN-γ), macrophage inflammatory protein (MIP)-1β and regulated upon activation normal T expressed and secreted (RANTES) in mRCC patient serum. Expression of these four cytokines within hepatocellular carcinoma correlated with a favorable prognosis. [26] This study of mRNA expression in hepatocellular carcinoma suggested that the anti-tumorigenic activities of these cytokines may result from the recruitment of tumor infiltrating lymphocytes to amplify the anti-tumor immune responses. Another study of RCC tumor tissue revealed that Th1 cytokine expression, e.g. Interferon gamma induced cytokines like IP-10, to be a favorable prognostic factor of survival after surgery. [16].

There were no detectable global differences in the gene expression profile of patients before (PRE) or after therapy (POST) that could be correlated to response. The absence of detectable treatment-related effects may be due to the timing of the POST sample used for gene expression profile analysis. It is possible that differences in gene expression are no longer detectable at 2 weeks following treatment, when POST PBLs were collected. Overall, these data suggest there are no lasting effects in the PBL gene expression profile of patients after treatment with DC-vaccine, IL-2 and IFN therapy.

However, further analyses revealed pathway-specific differences in responders and non-responders. We observed an increase in gene expression pathways that reflected more transcriptional activity and enhanced T-cell responses and less co-stimulation in responding patients, whereas non-responding patients had more active immunosuppressive pathways. Our results confirm that inhibitory pathways impact disease state and underscore the importance of regulating these pathways while concomitantly stimulating effector T cells for maximum clinical benefit.

Notably, in addition to up-regulation of genes associated with lymphocyte activation pathways in responders, the CTLA4 gene was less up-regulated in responders. We have previously shown that the number of T_REG_ cells induced by treatment correlates with response in this cohort of patients and now we demonstrate that the level of CTLA4 gene expression is altered and relates to response as well. [36] CTLA4 manifold control of tolerance is complex and involves competition with co-stimulatory molecules, dysregulation of the immunological synapse, inhibition of inflammatory cytokines, and preservation of CD4^+^ T_REG_ cell function. [37] Therapies with anti-CTLA4 blocking antibodies have some modest benefit in cancer patients. [38], [39] Taken together with our data, these studies suggest that suppressing CTLA4 signaling may be necessary but not sufficient for robust clinical response in cancer patients.

Gene expression of PBLs for responding patients also revealed an up-regulation of the HIFα and Flt3 pathways, and cluster analysis of genes in these pathways could discriminate NR from R patients. Evidence has been provided that hypoxia may down-regulate T-cell functions and an up-regulation of HIFα in lymphocytes may contribute to more vigorous anti-tumor effect at the hypoxic tumor microenvironment. [40] Flt3 is a tyrosine-kinase receptor involved in the early stages of development of hematopoietic cells. Flt3 is critical in the recovery of both B and T cells following myeloablative therapy, and can promote myeloid dendritic cell differentiation. [41]–[43] In contrast to its role in hematopoietic malignancies, an increase in genes of the Flt3 pathway in responding patients suggests that the induction of lymphopoiesis is an important step in stimulating anti-cancer immune responses in solid tumors. Interestingly, other studies have found that HIFα deficiency interferes with B and T cell function, [44] while HIFα expression in immune cells promotes cell survival and function. [45] These pathways may be useful biomarkers to predict outcome if confirmed in larger prospective immunotherapy studies.

Our goal of finding a predictive gene signature as a diagnostic tool that correlates with clinical outcome may have been limited by the small size of our study and the variation in the dataset due to patient individuality. Even though multiple studies have shown that expression profiling of the tumor itself can in principle be used to develop classifiers that allow prediction of prognosis or therapeutic responses, [46], [47] it has been very difficult to translate gene expression data into the clinic. [48], [49] As have many others, our study can distinguish between cancer patients and controls, [8]–[10], [50], [51] but the use of expression profiling of PBL/PBMC of patients as a surrogate biomarker in cancer will require much more work e.g. gene signature monitoring under therapy in the setting of much larger studies.

Nevertheless, we were able to observe differential regulation of genes enriched in immune activation and regulatory pathways in responding and non-responding patients upon immunotherapy. This type of analysis remains promising for ultimately identifying subpopulations of patients with unique potential to benefit from specific targeted therapies. Knowing the disposition of each patient’s immune system to activate regulatory or inflammatory pathways upon immune therapy will select candidate patients for e.g. a therapy with CTLA4-antagonist and IL2. Determining each patient’s immune gene profile before therapy might guide the decision for using immune therapy after a reasonable risk-benefit assessment and therefore enhance therapeutic responses to anticancer-immunotherapy by bolstering anti-tumor immune responses.

## Supporting Information

Figure S1 **Supervised hierarchical clustering analysis for T-cell and B-cell receptor associated genes of all arrays was performed.** Patients and healthy controls form distinct clusters. This supports the finding of altered T- and B-cell signaling and activation in mRCC patients.
(TIF)

Figure S2 **Genes that were differentially regulated between mRCC patients pre and post-treatment (based on p<0.05, logFC >1,4) were selected for clustering analysis that is displayed as a heatmap.** (green means low expression of a specific gene, red means high expression of a specific gene) This supervised analysis shows no distinct grouping of patient PBL gene signatures PRE and POST immunotherapy.
(TIF)

Figure S3 **Supervised hierarchical clustering of responding versus non-responding patients (based on p<0.05, logFC >1,4) is displayed as a heatmap for pre-treatment (3A) and post-treatment (3B) PBLs. No distinct clustering of the two groups occurs.**
(TIF)

Figure S4 **Comparison of treatment related changes in gene expression for responding versus non-responding patients (POST minus PRE) and analysis with IPA.** Immune therapy leads to decreased expression of CTLA4 related genes in responding subjects.
(TIF)

Figure S5 **Peripheral blood serum concentrations for the cytokine Interferon inducible factor 10, IP-10 for healthy controls and mRCC patients PRE and POST treatment as a group and split based on response (n: 5 healthy controls, 8 mRCC: 4 R PRE, 4 NR PRE, 4 R POST, 4 NR POST).** RCC patients show significantly higher levels of IP-10. Responders have higher levels of IP10 PRE (ns, p = 0.08). IP-10 serum levels increase with immune therapy in R and NR. This increase is significant for the patient group (p<0.01) and for the smaller group of responding patients (p<0.05).
(TIF)

Figure S6 **Correlation between the Fold Changes of 132 RCC-associated transcripts identified in PBMCs by Twine et al (1), and the corresponding fold change for our PBL based data set.** The analysis revealed no similarities in gene expression levels which may be due to additional monocyte derived cell types in the PBMCs.
(TIF)

Table S1 **Primer assays for RT-PCR.**
(DOCX)

Table S2 **Validation of index genes with RT-PCR. Differences in gene expression for patients vs. healthy controls as well as PRE vs. POST seen in microarray analysis could be confirmed with RT-PCR.** Negative fold changes in microarray analysis as seen on the right side for PRE vs. POST correlate to corresponding RT-PCR fold changes. Two methods of fold change calculation (difference vs. ratio) had to be taken into account. Same RNA specimens were used for microarray analysis and RT-PCR. A clear correlation between relative expression values in RT-PCR with microarray analysis could be shown for all tested genes.
(DOCX)
