# Supplementary figures and images for: Gene Expression Profile of Peripheral Blood Lymphocytes from Renal Cell Carcinoma Patients Treated with IL-2, Interferon-α and Dendritic Cell Vaccine
Source: PLoS One. 2012 Dec 3;7(12):e50221. doi: 10.1371/journal.pone.0050221 (PMC3513309; doi:10.1371/journal.pone.0050221)

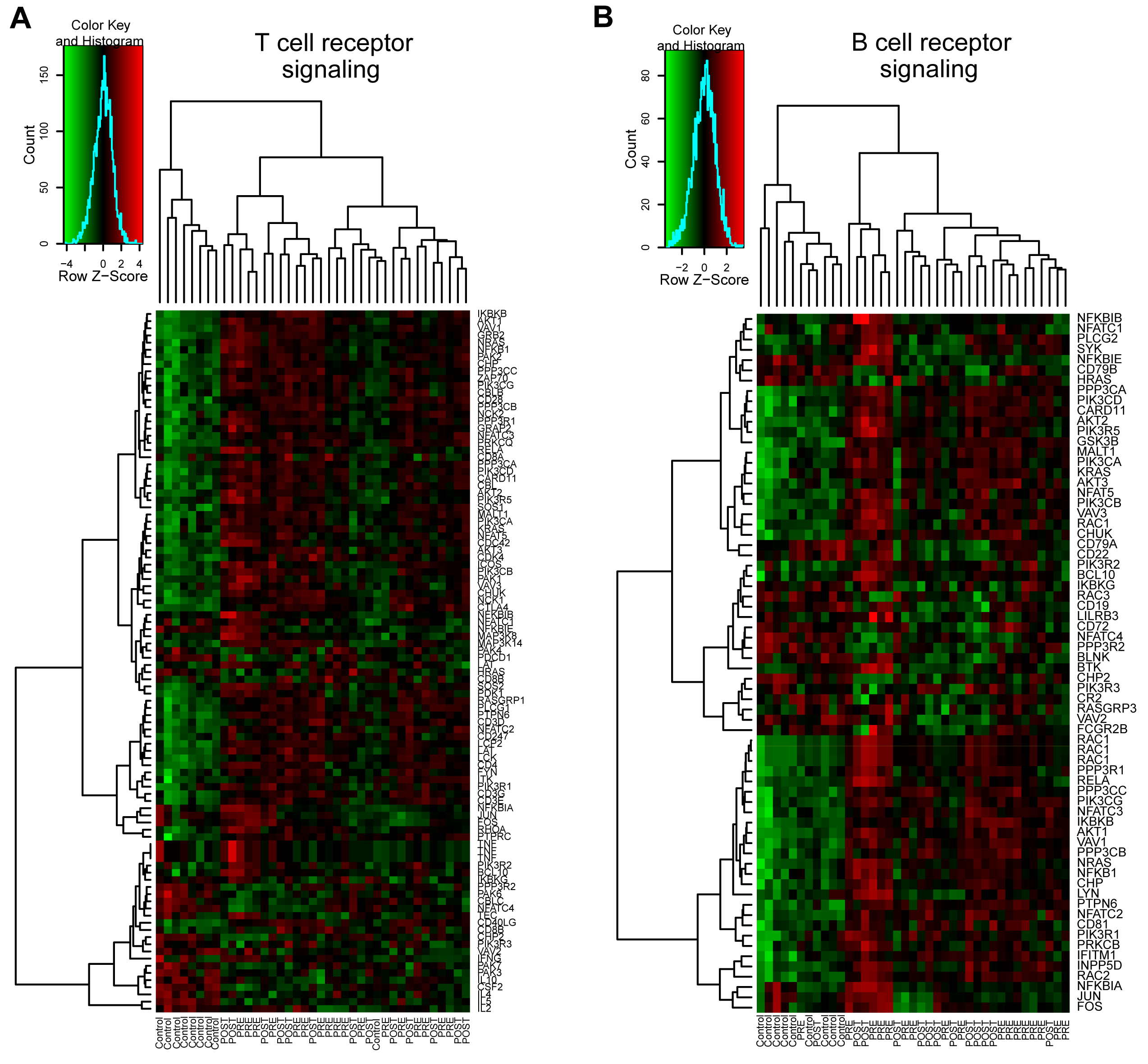

Supplement: Figure S1 — Supervised hierarchical clustering analysis for T-cell and B-cell receptor associated genes of all arrays was performed. Patients and healthy controls form distinct clusters. This supports the finding of altered T- and B-cell signaling and activation in mRCC patients. (TIF) [file pone.0050221.s001.tif]

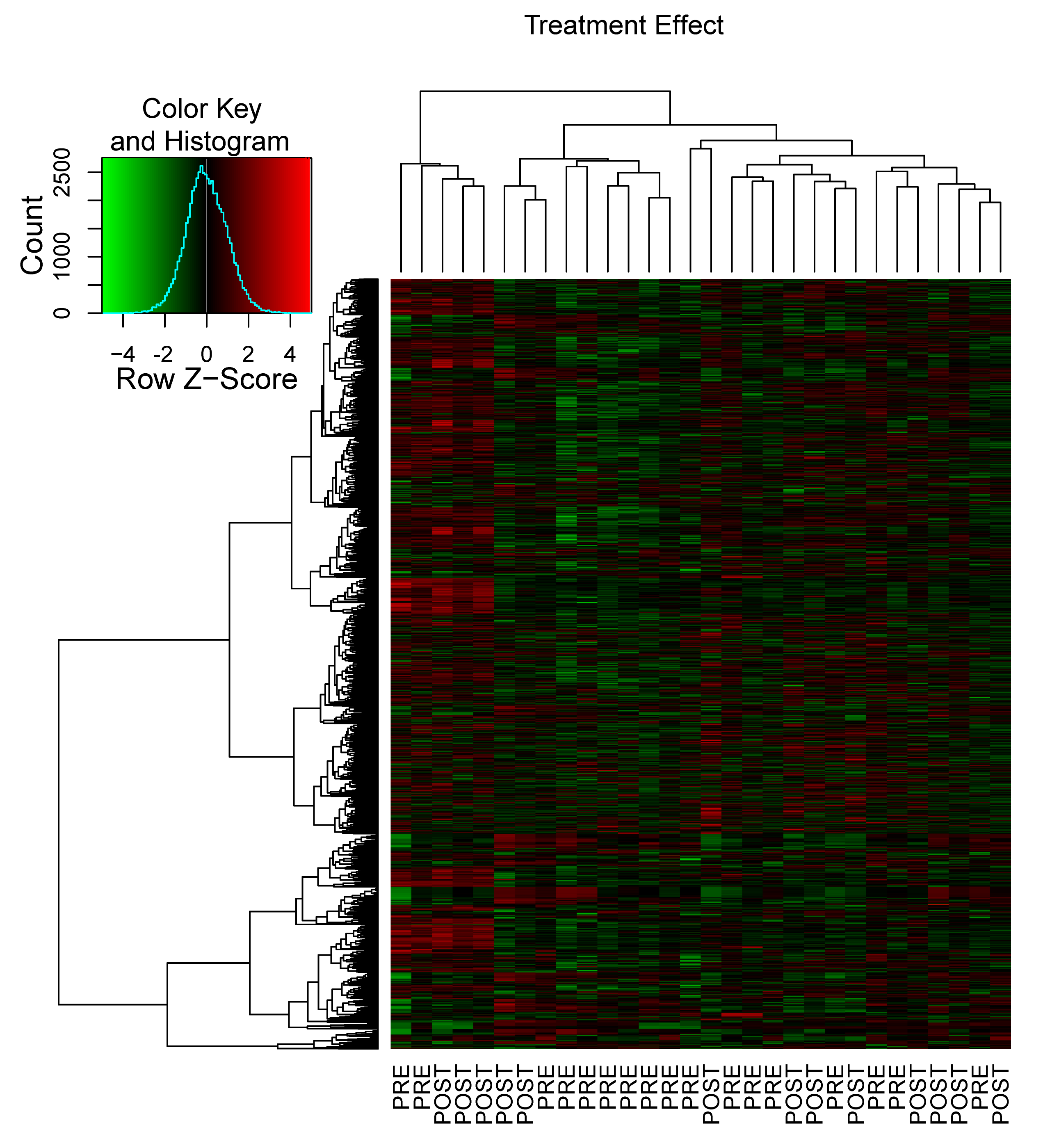

Supplement: Figure S2 — Genes that were differentially regulated between mRCC patients pre and post-treatment (based on p<0.05, logFC >1,4) were selected for clustering analysis that is displayed as a heatmap. (green means low expression of a specific gene, red means high expression of a specific gene) This supervised analysis shows no distinct grouping of patient PBL gene signatures PRE and POST immunotherapy. (TIF) [file pone.0050221.s002.tif]

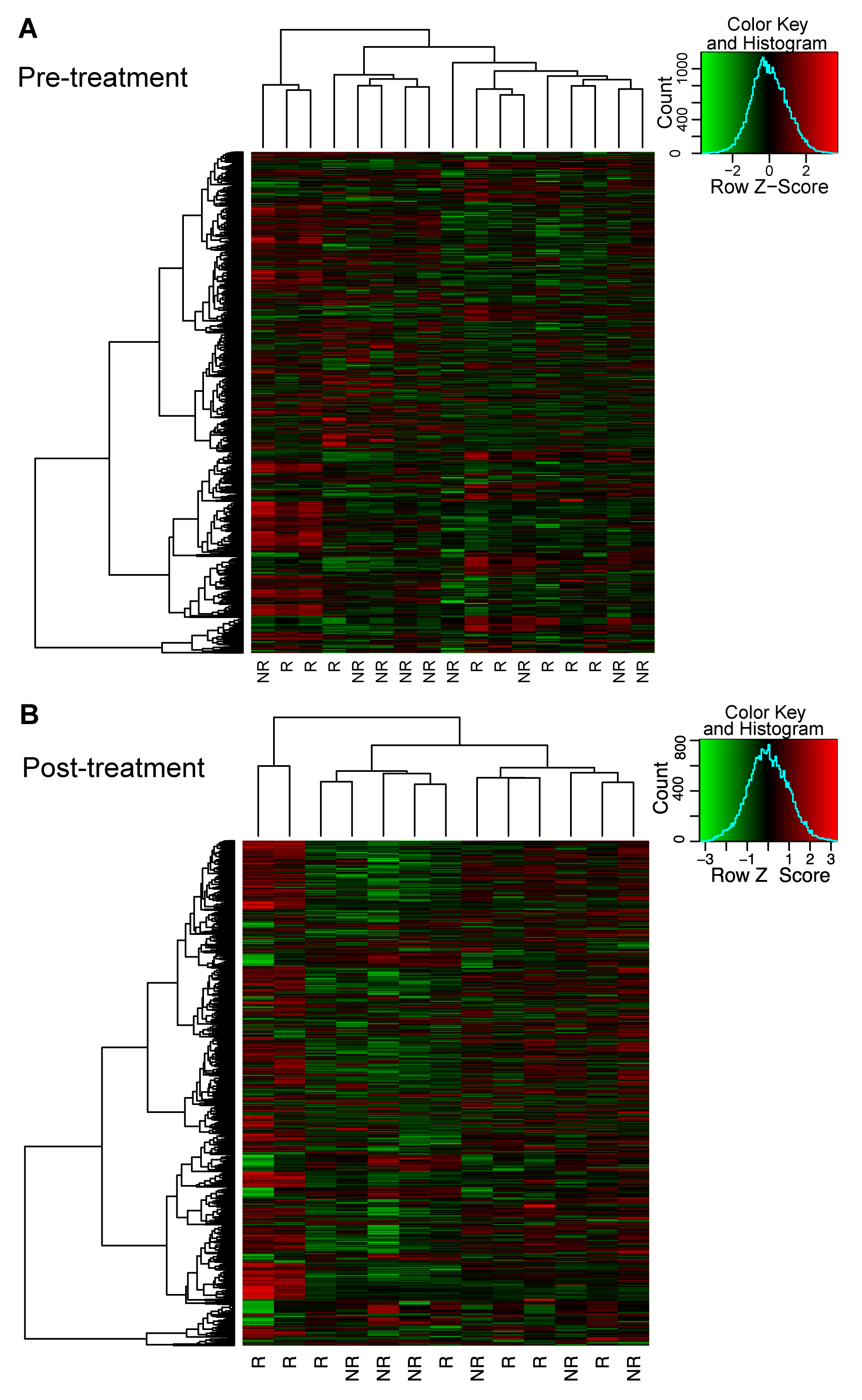

Supplement: Figure S3 — Supervised hierarchical clustering of responding versus non-responding patients (based on p<0.05, logFC >1,4) is displayed as a heatmap for pre-treatment (3A) and post-treatment (3B) PBLs. No distinct clustering of the two groups occurs. (TIF) [file pone.0050221.s003.tif]

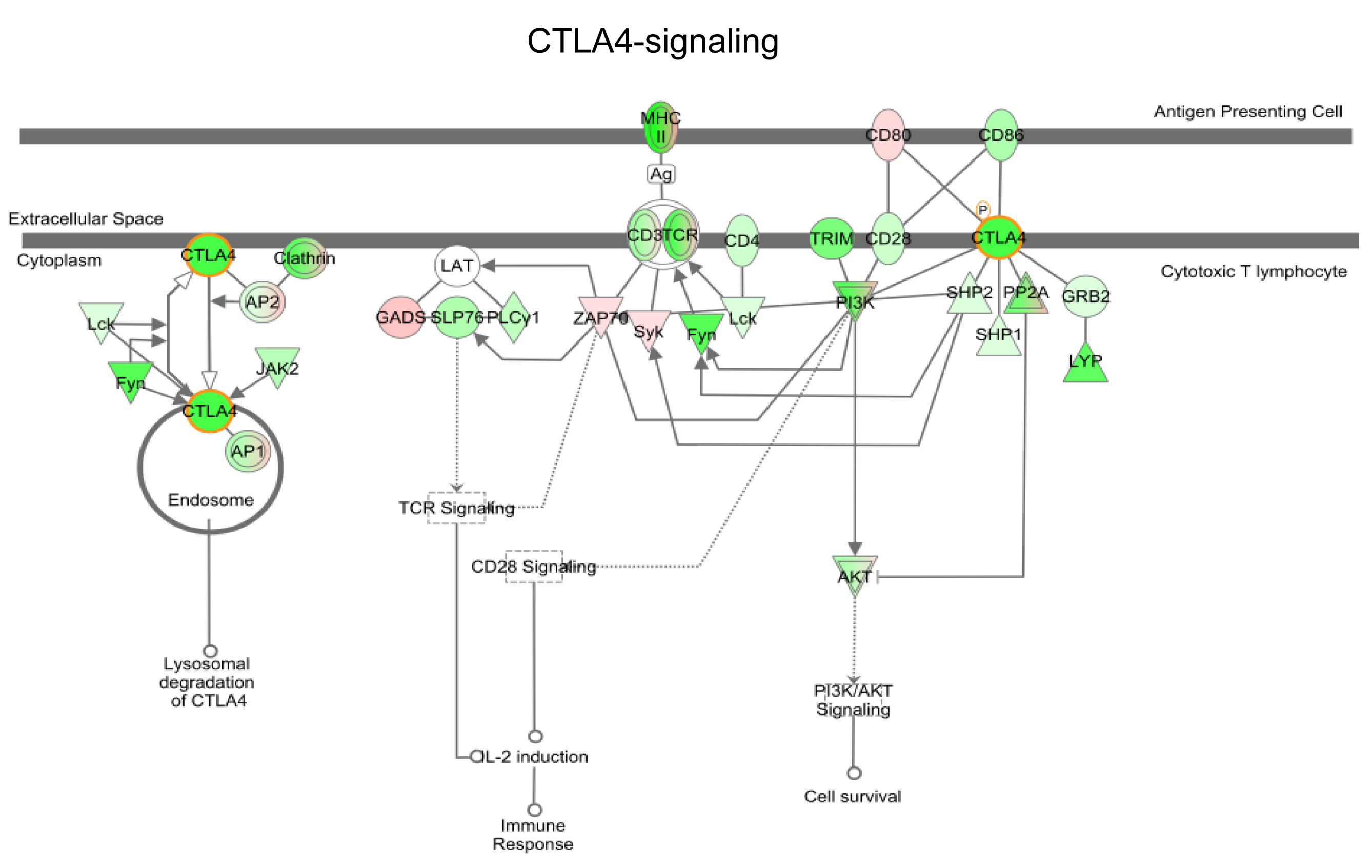

Supplement: Figure S4 — Comparison of treatment related changes in gene expression for responding versus non-responding patients (POST minus PRE) and analysis with IPA. Immune therapy leads to decreased expression of CTLA4 related genes in responding subjects. (TIF) [file pone.0050221.s004.tif]

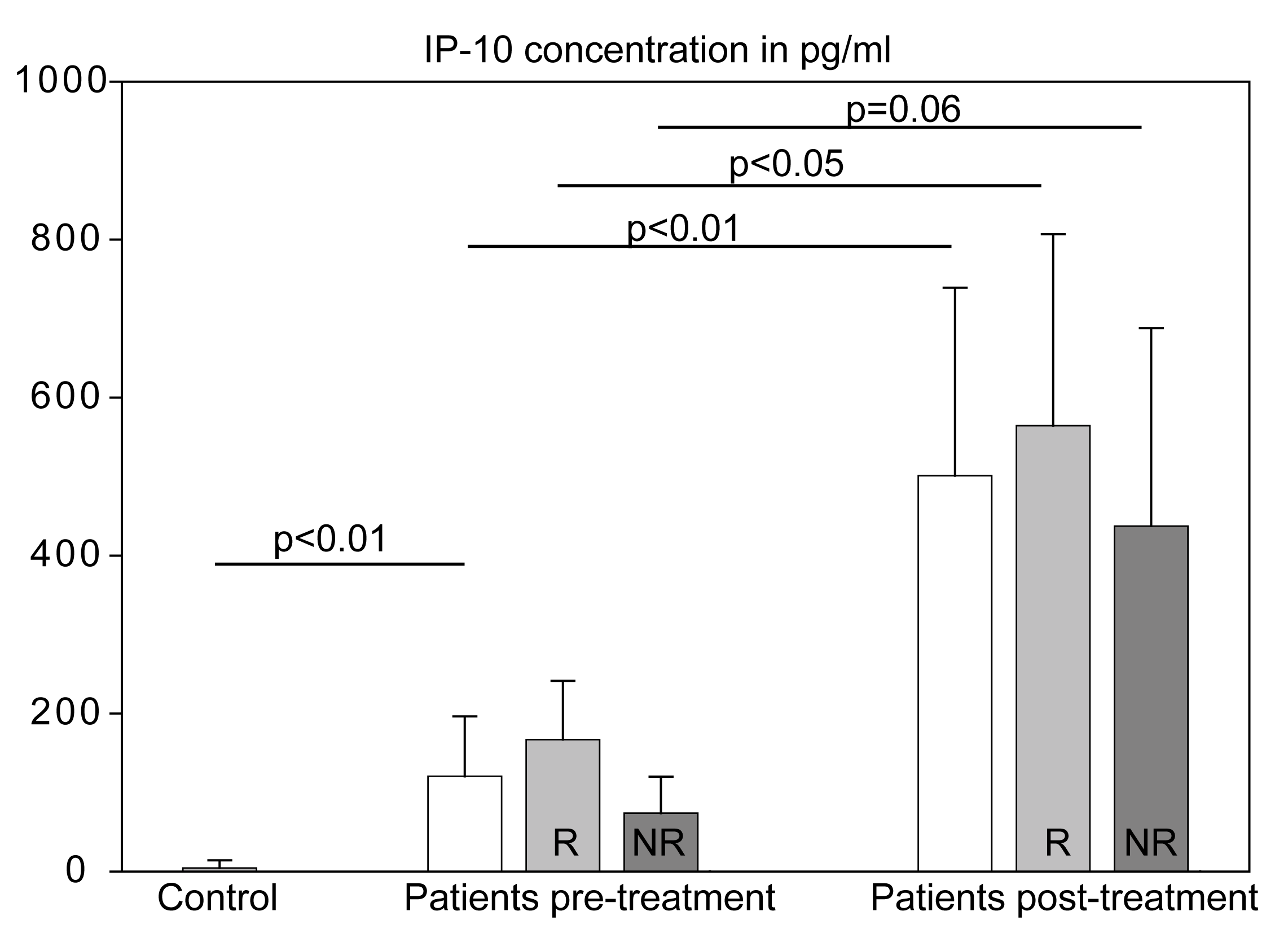

Supplement: Figure S5 — Peripheral blood serum concentrations for the cytokine Interferon inducible factor 10, IP-10 for healthy controls and mRCC patients PRE and POST treatment as a group and split based on response (n: 5 healthy controls, 8 mRCC: 4 R PRE, 4 NR PRE, 4 R POST, 4 NR POST). RCC patients show significantly higher levels of IP-10. Responders have higher levels of IP10 PRE (ns, p = 0.08). IP-10 serum levels increase with immune therapy in R and NR. This increase is significant for the patient group (p<0.01) and for the smaller group of responding patients (p<0.05). (TIF) [file pone.0050221.s005.tif]

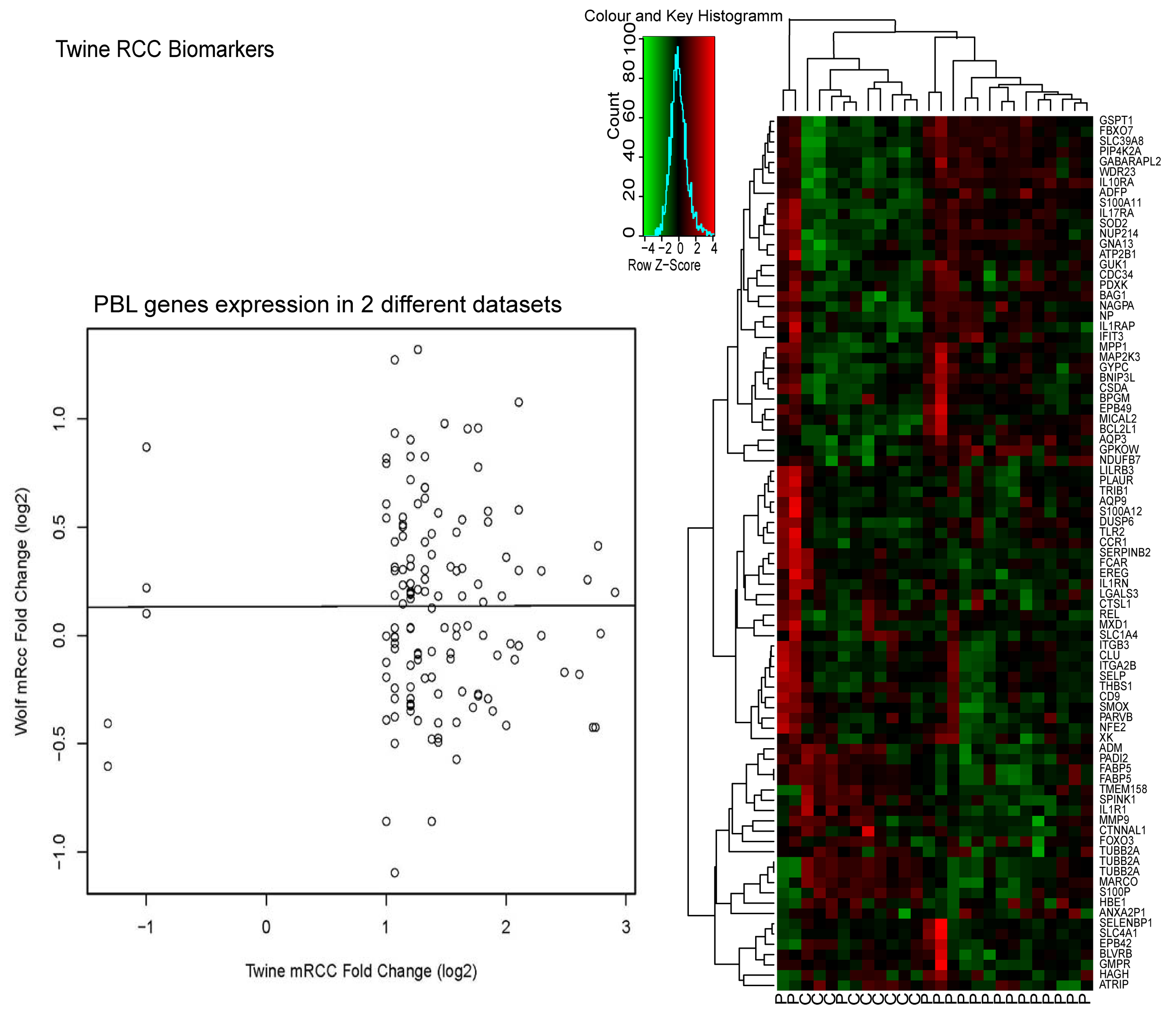

Supplement: Figure S6 — Correlation between the Fold Changes of 132 RCC-associated transcripts identified in PBMCs by Twine et al (1), and the corresponding fold change for our PBL based data set. The analysis revealed no similarities in gene expression levels which may be due to additional monocyte derived cell types in the PBMCs. (TIF) [file pone.0050221.s006.tif]
