# Supplementary material for: Gene Expression Profile of Peripheral Blood Lymphocytes from Renal Cell Carcinoma Patients Treated with IL-2, Interferon-α and Dendritic Cell Vaccine
Source: PLoS One. 2012 Dec 3;7(12):e50221. doi: 10.1371/journal.pone.0050221 (PMC3513309; doi:10.1371/journal.pone.0050221)
Supplement: Table S1 — Primer assays for RT-PCR. (DOCX) [file pone.0050221.s007.docx]

Table S1: Primer assays for RT-PCR

|  | Abbreviation | QT-Assay Nr. | EntrezGeneID |
| --- | --- | --- | --- |
| Toll like receptor 10 | TLR 10 | QT0020547 | 81793 |
| Kynurenine 3-monooxygenase | KMO1SG | QT00062405 | 8564 |
| Interleukin 2 receptor alpha | IL-2RA | QT00040754 | 3559 |
| Interferon-induced protein 44-like | IFI44L 1 | QT00051457 | 10964 |
| CD40 ligand | CD40LG | QT00000343 | 959 |
| Chemokine ligand 3 | CCL3-2 | QT01008063 | 6348 |
| T-cell receptor associated transmembrane adaptor 1 | TRAT1-1 | QT00024388 | 50852 |
| Chemokine (C-X3-C motif) receptor 1 | CX3CR1 | QT00203434 | 1524 |
| Tumor necrosis factor (ligand) superfamily, member 10 | TNFSF10 | QT00079212 | 8743 |
| Forkhead box P3 | FOXP3 | QT00048286 | 50943 |
| Glycerinaldehyd-3-phosphat-Dehydrogenase | GAPDH | QT01192646 | 2597 |
