# Supplementary material for: Gene Expression Profile of Peripheral Blood Lymphocytes from Renal Cell Carcinoma Patients Treated with IL-2, Interferon-α and Dendritic Cell Vaccine
Source: PLoS One. 2012 Dec 3;7(12):e50221. doi: 10.1371/journal.pone.0050221 (PMC3513309; doi:10.1371/journal.pone.0050221)
Supplement: Table S2 — Validation of index genes with RT-PCR. Differences in gene expression for patients vs. healthy controls as well as PRE vs. POST seen in microarray analysis could be confirmed with RT-PCR. Negative fold changes in microarray analysis as seen on the right side for PRE vs. POST correlate to corresponding RT-PCR fold changes. Two methods of fold change calculation (difference vs. ratio) had to be taken into account. Same RNA specimens were used for microarray analysis and RT-PCR. A clear correlation between relative expression values in RT-PCR with microarray analysis could be shown for all tested genes. (DOCX) [file pone.0050221.s008.docx]

| **Patients pre vs. healthy control** | | | | **Patients pre vs. patients post** | | | |
| --- | --- | --- | --- | --- | --- | --- | --- |
|  | **Microarray** | **RT-PCR** | |  | **Microarray** | **RT-PCR** | |
|  | **Fold Change (difference)** | **Delta delta CT** | **Fold change (ratio)** |  | **Fold Change (difference)** | **Delta delta CT** | **Fold change (ratio)** |
| **Gene** |  |  |  | **Gene** |  |  |  |
| CX3CR1 | 3.70 | -1.84 | 3.59 | TLR10 | -1.37 | 0.73 | 0.60 |
| TNFSF10 | 3.68 | -1.80 | 3.49 | CCL3 | 1.30 | -1.75 | 3.35 |
| IFI44L | 3.16 | -1.21 | 2.32 | FOXP3 | 1.17 | -1.02 | 2.03 |
| CD40L | 2.45 | -1.81 | 3.51 | KMO | -1.27 | 0.87 | 0.54 |
| TRAT1 | 2.33 | -1.10 | 2.15 | IL2-RA | 1.57 | -0.40 | 1.31 |

Table S2: Validation of index genes with RT-PCR. Differences in gene expression for patients vs. healthy controls as well as PRE vs. POST seen in microarray analysis could be confirmed with RT-PCR. Negative fold changes in microarray analysis as seen on the right side for PRE vs. POST correlate to corresponding RT-PCR fold changes. Two methods of fold change calculation (difference vs. ratio) had to be taken into account. Same RNA specimens were used for microarray analysis and RT-PCR. A clear correlation between relative expression values in RT-PCR with microarray analysis could be shown for all tested genes.
